# Altered *CSMD1* Expression Alters Cocaine-Conditioned Place Preference: Mutual Support for a Complex Locus from Human and Mouse Models

**DOI:** 10.1371/journal.pone.0120908

**Published:** 2015-07-14

**Authors:** Jana Drgonova, Donna Walther, Sulabh Singhal, Kennedy Johnson, Brice Kessler, Juan Troncoso, George R. Uhl

**Affiliations:** 1 Molecular Nuropsychiatry Research Branch, NIH-IRP, NIDA, Baltimore, Maryland, United States of America; 2 Division of Neuropathology, Johns Hopkins School of Medicine, Baltimore MD, United States of America; 3 Office of Research & Development, New Mexico VA Healthcare System, Albuquerque, NM, United States of America; University of Leicester, UNITED KINGDOM

## Abstract

The CUB and sushi multiple domains 1 (*CSMD1*) gene harbors signals provided by clusters of nearby SNPs with 10^-2^ > p > 10^-8^ associations in genome wide association (GWAS) studies of addiction-related phenotypes. A *CSMD1* intron 3 SNP displays p < 10^-8^ association with schizophrenia and more modest associations with individual differences in performance on tests of cognitive abilities. *CSDM1* encodes a cell adhesion molecule likely to influence development, connections and plasticity of brain circuits in which it is expressed. We tested association between *CSMD1* genotypes and expression of its mRNA in postmortem human brains (n = 181). Expression of *CSMD1* mRNA in human postmortem cerebral cortical samples differs 15–25%, in individuals with different alleles of simple sequence length and SNP polymorphisms located in the gene’s third/fifth introns, providing nominal though not Bonferroni-corrected significance. These data support mice with altered CSMD1 expression as models for common human CSMD1 allelic variation. We tested baseline and/or cocaine-evoked addiction, emotion, motor and memory-related behaviors in +/- and -/- *csmd1* knockout mice on mixed and on C57-backcrossed genetic backgrounds. Initial *csmd1* knockout mice on mixed genetic backgrounds displayed a variety of coat colors and sizable individual differences in responses during behavioral testing. Backcrossed mice displayed uniform black coat colors. Cocaine conditioned place preference testing revealed significant influences of genotype (p = 0.02). Homozygote knockouts displayed poorer performance on aspects of the Morris water maze task. They displayed increased locomotion in some, though not all, environments. The combined data thus support roles for common level-of-expression *CSMD1* variation in a drug reward phenotype relevant to addiction and in cognitive differences that might be relevant to schizophrenia. Mouse model results can complement data from human association findings of modest magnitude that identify likely polygenic influences.

## Introduction

The gene that encodes the CUB and sushi multiple domains 1 protein (*CSMD1*) has been identified in case *vs* control genome-wide association (GWAS) samples for addiction- related phenotypes that include extensive use of an addictive substance, vulnerability to develop dependence on an addictive substance, ability to quit smoking and time from initiation of tobacco use to development of nicotine dependence. The modest association signals identified in these studies come from clusters of nearby single nucleotide polymorphisms (SNPs) with 10^−2^ > p > 10^−8^ association that often do not survive conservative Bonferroni corrections [1,2,3,4,5,6,7,8,9,10,11,12,13]. The distribution of these signals in several parts of the CSMD1 gene is consistent with substantial disease-related allelic heterogeneity.

*CSMD1* has also been identified by several analyses of GWAS data and/or candidate gene studies for vulnerability to schizophrenia and clusters of schizophrenic symptoms [14,15,16,17,18,19]. Association with the *CSMD1* intron 3 SNP rs10503253 is among the most studied. This or nearby SNPs have also been associated, at least modestly, with individual differences in cognitive abilities in normal and in schizophrenic samples [20,21,22,23,24]. Nominal p values were 3 x 10^−3^–1 x 10^−4^ for tests of IQ, strategy formation, planning, set shifting and problem solving in Greek military conscripts [20], 0.01–0.04 for verbal IQ, performance IQ and/or mnemonic measures in Irish and/or German schizophrenics and controls [23] and 2×10^−6^ with “memory cognition” in Chinese schizophrenics and controls [24].Three common *CSMD1* missense variants with minor allele frequencies > 0.03 are listed in databases. However, all are distant from intron 3 (http://www.ncbi.nlm.nih.gov/projects/SNP/snp_ref.cgi?geneId=64478). Increased frequencies of *CSMD1* copy number variants are reported in individuals with mild cognitive impairment and with dementia [25]. However, little reported data links specific human *CSMD1* genomic variants to specific molecular or behavioral phenotypes relevant to particular aspects of addiction or schizophrenia.

Features of *CSMD1*’s neurobiology make its variants attractive candidates to contribute to individual differences in vulnerability to addiction and in mnemonic processes. The *CSMD1* gene encodes a single transmembrane domain protein likely to alter the development and maintenance of connections between expressing neurons. Abundant CSMD1 immunoreactivity is found at the growth cones of cultured neurons [26]. Ventral midbrain neurons implicated in reward, likely to be dopaminergic, prominently express CSMD1 mRNA, as do hippocampal neurons implicated in mnemonic processes (http://mouse.brain-map.org/experiment/ivt?id=69608130&popup=true). Results from studies of *csmd1* knockouts on mixed genetic backgrounds *(see below)* also support interesting phenotypes [21,27].

These neurobiologic, genetic and genomic data nominate *CSMD1* as a candidate for studies that seek: a) influences of common human allelic variation on *CSMD1* expression, b) influences of variation in CSMD1 expression on responses to rewarding addictive substances in mouse models, c) influences of variation in *CSMD1* expression on cognitive phenotypes that may model features relevant to schizophrenia and d) comparisons with other physiological and behavioral differences in mice with altered *csmd1* expression.

We now report studies of *CSMD1* expression in human postmortem cerebral cortical samples that identify nominally-significant associations between levels of CSMD1 expression and *CSMD1* genomic markers, including those that lie near the schizophrenia-associated SNP rs10503253, though these associations do not survive conservative Bonferroni corrections. We describe our initial data from the *csmd1* knockouts on mixed genetic backgrounds and report the variability that differences in genetic background among these mice appears to provide. We then describe more extensive results from “*csmd1*” knockout mice backcrossed onto a C57 background for 5 generations. We study results of tests that include cocaine conditioned place preference (CPP), one of the most commonly used and heavily validated mouse tests for drug reward/reinforcement [28]. We demonstrate that mice with altered CSMD1 expression display overall differences in cocaine CPP, although their locomotion is influenced by modest to moderate doses of cocaine in ways similar to those of wildtype mice. There is modestly increased locomotion in homozygous knockouts. We identify alterations in Morris water maze testing in homozygous backcrossed *csmd1* knockouts, and discuss the potential implications of these data for the *CSMD1* associations with cognitive differences in schizophrenia, in normal populations, and for our CPP data from heterozygous and homozygous mice. We note ways in which these data enhance our confidence that CSMD1 variation and thus the neuronal properties and connections that CSMD1 modulates play roles in addiction phenotypes and in cognition-related phenotypes that are of likely relevance for schizophrenia.

## Materials and Methods

### Common human allelic *CSMD1* sequence variation was sought

Common human allelic *CSMD1* sequence variation was sought by searches of dbSNP (http://www.ncbi.nlm.nih.gov/SNP/) and the Toronto database for structural/copy number variation (http://dgvbeta.tcag.ca/dgv/app/home?ref=NCBI36/hg18). Genetic *cis* influences on levels of CSMD1 expression were sought by studies of CSMD1 mRNA and DNA in rapidly-frozen autopsy samples of frontal cortex of European American individuals who died without brain disease. All brain samples were supplied anonymously from tissue banks at the University of Maryland (http://medschool.umaryland.edu/btbank/) and Johns Hopkins University (http://pathology.jhu.edu/department/services/consults/neuropath.cfm). RNAs were prepared with the RNeasy lipid tissue mini kits (Qiagen), cDNA was synthesized with SuperScript III First Strand Synthesis Supermix (Invitrogen) and levels of mRNAs were assessed by quantitative RT-PCR using SybrGreen master mix (Applied Biosystems), conditions from the manufacturer’s protocol and oligonucleotide primers (*sequences available from authors on request*) that targeted the dominant long CSMD1 mRNA isoform (http://www.ncbi.nlm.nih.gov/IEB/Research/Acembly/av.cgi?db=human&q=CSMD1) and the reference genes glyceraldehyde-3-phosphate dehydrogenase (GAPDH), hypoxanthine phosphoribosyltransferase 1 (HPRT1) and ubiquitin C (UBC).

DNA was extracted from brain samples using Qiagen kits [29], and subjected to multiplexed SNP genotyping using Sequenom panels and oligonucleotides (S1 Table) for 38 SNPs distributed through the gene. The simple sequence repeat that is annotated as rs71534387 was amplified using oligonucleotide primers, polymerase chain reaction conditions 1X PCR Gold buffer (Invitrogen), 0.8 mM dNTP mix, 1.5 mM MgCl_2_, 0.4μM forward and reverse primers and 0.25 units Amplitaq Gold enzyme (Invitrogen). Amplimers and oligonucleotides provided clear peaks every 3 bp after separation using an Applied Biosystems 3730xl instrument with Liz500 size standard (performed by Genewiz, Inc.). Peaks were analyzed using Peak Scanner software and genotypes determined for each DNA sample.

### Mouse models

Initial constitutive *csmd1* homozygous knockout (KO), heterozygous knockout and littermate wildtype animals were produced by heterozygote x heterozygote crosses from mice that were originally created by Lexicon pharmaceuticals, distributed by Taconic Farms (TF0137) and described by others [21,27]. Embryonic stem cells derived from 129SvEvBrd mice replaced a 1,070 bp genomic sequence of the *csmd1* exon 1–intron 1 junction with a LacZ/Neo selection cassette expressed in frame with the start of the CSMD1 protein-coding sequence. The “mixed background” mice derived from these ES cells were maintained on mixed genetic backgrounds that included 129 and B6 ancestries (the exact B6 substrain unknown) as reflected by varying coat colors (black, agouti, and white individuals) [21,27]. Following initial testing and identification of substantial mouse to mouse variability, these mixed background *csmd1* KO mice were backcrossed to C57Bl/6J mice for 4 generations and then to Tg(Thy1-EGFP)MJrs mice (C57Bl/6J mice expressing eGFP under control of the *Thy1* promoter) for the fifth generation, so that less than 2% of the initial 129 DNA was present. These backcrossed mice, termed *csmd1* mice here, of both sexes were tested at 118 ± 49 days of age. All mouse breeding, care and experimentation was approved by the NIDA-IRP Animal Care and Use Committee.

### Mouse behavioral studies

#### Motor

*Muscle strength/motor persistence test*: 9–10 mice of each genotype, half of them males and half females were tested to determine the time during which they could support their weight by holding onto a wire screen (5 mm hardware cloth) mounted 20 cm above and parallel to the countertop. The test was terminated at 120 sec, or when the mouse fell.

*Motor coordination and learning* in the same cohort of animals were measured on an accelerating rotarod cylinder on which the mice had to maintain their balance to prevent falling. The animals were tested over three consecutive days with one test per day. The starting speed of the rotating cylinder was 4 rpm and it gradually increased to 40 rpm over 5 min, which was the cut off time.

#### Cocaine effects

*Reward*: *cocaine conditioned place preference* was assessed in 255 naive animals, 25–26 and 13–16 subjects/genotype for 10 mg/kg and other doses, respectively, half males and half females as in [30,31]. Mice were 12 ± 3 weeks old and weighed on the pretest day to determine dose. Two 20 cm x 20 cm compartments, one with a wire-mesh floor and the other with corn cob bedding, were separated by a Plexiglas divider. In two 20 min pre-tests, subjects had access to both sides of the apparatus through a 5 cm opening in the divider. Four 20 min conditioning trials were conducted over the next 2 days during which subjects were confined to one side of the apparatus. Mice were injected with cocaine prior to confinement in the initially non-preferred compartment and with saline prior to confinement in the initially-preferred compartment. Half of the mice received cocaine/initially non preferred and half received saline/initially preferred as their first drug/environmental pairings on the first conditioning day; the order for each mouse was reversed on the second day. Control subjects received saline injections prior to separate confinements on each side. 18 hours after the last conditioning session (48 hours after the first conditioning session), subjects were again given access to both sides of the apparatus for a 20 min post-test. The preference score was calculated as the difference between time spent on the drug-paired side during the post-test and the average time spent on the drug-paired side during the pre-tests.

*Locomotion* was recorded: a) in 42 x 42 cm dark, sound attenuated boxes to which the mice had not been previously exposed, for 60 min trials (n = 10-11/genotype) and b) during the 20 min pretest (in both halves of the 20 x 40 conditioning apparatus), conditioning (20 x 20 cm half of the apparatus) and test (20 x 40 cm) sessions. Data was thus obtained from untreated, saline treated, and mice sampled before treatment, after one treatment and after the second treatment with cocaine. Total distance traveled was calculated from infrared beam breaks by an Optovarimax ATS System [31].

*Memory and learning* were evaluated in Morris water maze testing [32] in 19–24 mice/genotype of the mice previously submitted to the hanging wire, rotarod or cocaine-conditioned place preference tests. A black 90 cm diameter pool was filled with room temperature water made opaque with white tempera paint. A 9 cm diameter platform was located in the center of one quadrant, visible for the first 6 trials and then hidden 0.5 cm below the water level for subsequent trials. Each trial lasted a maximum of 60 seconds and was followed by a 15 second rest period on the platform. After two trials, mice were returned to their home cages for about 4 hours and then given an additional 2-trial session so that they received a total of 4 trials per day. For each trial, the latency to reach the platform was recorded. Three days after acquisition, defined by an average latency less than 10 seconds, the platform was removed and a 60 second probe trial was conducted. The data from the probe trial was analyzed with Ethovision software (Noldus, Netherlands), which also determined the path of the subject in the pool and the time spent in each quadrant.

### Statistical analyses

Analyses of mouse data used PASW statistics 18 (SPSS) and t tests (Excel). Analyses of variance and covariance (ANOVA and ANCOVA) used between subjects factors of genotype sex, and dose, age as a cofactor, and within-subjects factors where appropriate including time for locomotion and trial day for Morris water maze. Analyses of human association data and assessment of correlations between genotypes at nearby SNPs used PLINK (pngu.mgh.harvard.edu/~purcell/plink). Normalized gene expression levels were compared between human major *vs* minor allele homozygotes using t tests. Bonferroni corrections were performed using (http://www.quantitativeskills.com/sisa/calculations/bonfer.php) with 0.6 and 0.8 correlations (r) between SNPs.

## Results

### Searches for common *CSMD1* variation related to addiction and/or schizophrenia

**Databases contained** no common *CSMD1* copy number/insertion/deletion variants, though rarer copy number variants that cover much of this large gene are documented. Only three missense variants (rs28455997, rs6558702 and rs11984691), which were distant from the most schizophrenia-associated SNP rs10503253, displayed minor allele frequencies > 0.03.

**Fig 1:**
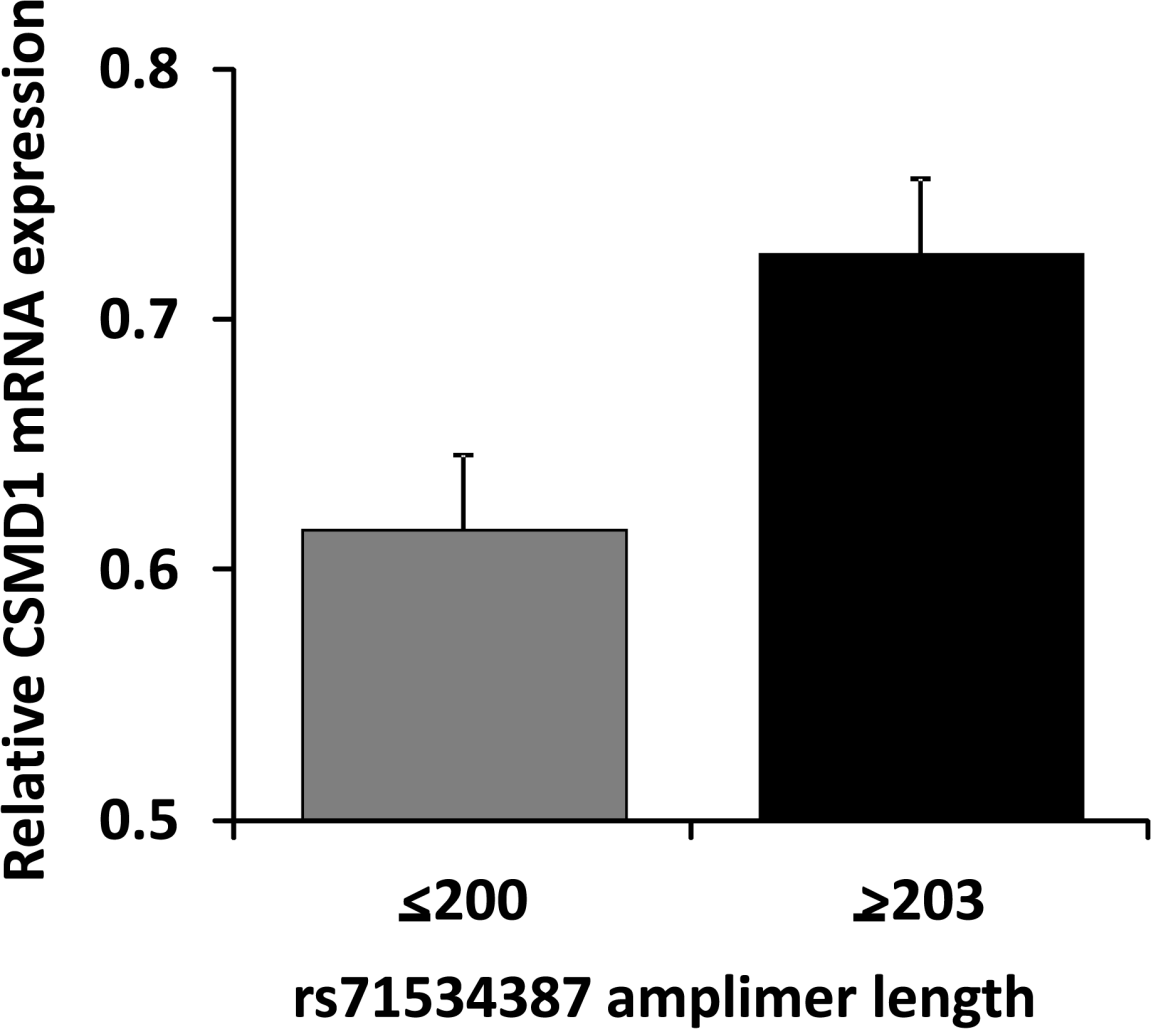
Differential expression of CSMD1 mRNA in cerebral cortical samples from individuals with different rs71534387 SSLP genotypes. Mean +/- standard error of the mean (SEM) of relative expression of CSMD1 mRNA in cerebral cortical samples of 72 EuAm individuals with no rs71534387 allele > 203 bp *vs* 96 individuals with at least one rs71534387 allele > 203 bp. Triplicate RT-PCR assays. Relative expression was determined by the mean of two CSMD1 amplimers in relation to the geometric mean of three control mRNAs from the same sample.

There was nominally-significant association between *CSMD1* genomic markers and levels of *CSMD1* mRNA expression. We detected *CSMD1* mRNA in RNA extracted from cerebral cortical specimens from each of the 181 individuals tested. Data from thirty nine genomic markers that displayed minor allele frequencies > 0.03 were analyzed. The *CSMD1* intron 3 trinucleotide repeat that is annotated as rs71534387 and the intron 5 SNP rs10503223 displayed the most robust associations (Fig 1; p = 0.03/0.02, respectively; t tests). The Bonferroni-corrected thresholds for two sided t tests are p < 0.012 and p < 0.024 with r = 0.6 and r = 0.8, respectively; these nominally-significant associations thus did not definitively surpass Bonferroni correction for the numbers of markers tested. These two markers displayed D’ values of 0.53 in individuals who donated the brain samples studied here. rs71534387 data comes from 104 individuals who were either hetero or homozygous for the 200 bp *vs* 206 bp alleles. These nominally-significant associations corresponded to 18/25% differences in levels of expression. Expression is lower in individuals with longer rs71534387 repeat lengths and in rs10503223 = G homozygotes. These differences in postmortem brain expression help to support heterozygous and/or homozygous *csmd1* knockout mice as models for common *CSMD1* allelic variation of relevance for addiction and, possibly, schizophrenia-related phenotypes. The schizophrenia-associated SNP rs10503253 itself did not provide the nominally-significant associations noted for the nearby rs71534387 trinucleotide repeat, however.

### Mice

#### Initial characterization of mice on mixed genetic backgrounds

Heterozygote x heterozygote crosses of mice on mixed genetic backgrounds supplied by Taconic produced offspring with genotypes in expected Mendelian ratios that displayed differences in coat colors; different mice had agouti, black or white coat colors. Initial tests of mice of these mixed genetic backgrounds revealed no significant influences of genotype on locomotion after injections of saline or 10 mg/kg cocaine doses (Table A in S2 Table; ANCOVA p = 0.729 and 0.650, respectively) or performance on the rotorod test of motor coordination/motor learning (Table B in S2 Table; ANCOVA effect of genotype p = 0.771).

#### Characterization of mice on C57Bl genetic backgrounds

We thus backcrossed *csmd1* knockout mice of the original mixed genetic background for 5 generations to mice with C57Bl/6J genetic backgrounds as noted above, producing backcrossed *csmd1* knockouts that we term *csmd1* knockouts in the rest of this paper. There was significance for the modest differences in weights for the knockout males but not for the females. Weights for male/female were wildtype: 28.6/21.3 g; heterozygotes 28.6/21.5 g and homozygotes 27.1/20.7 g, n = 39–45/genotype, (Table C in S2 Table; ANCOVA with age as covariate p = 0.019 for males and 0.237 for females). Since there were no significant sex * genotype interactions, the small, sex-specific differences did not provide effects on behavioral results of the knockout.

These backcrossed mice were subjected to a number of physiologic, pharmacological and behavioral tests. The *csmd1* +/- and-/- knockouts displayed no evidence for gross alterations in motor function. They were similar to wildtype littermates in screen hang time and rotarod testing (Tables D and E in S2 Table; ANCOVA p values for effects of genotype 0.346 and 0.402, respectively).

*csmd1* knockout mice failed to display significant differences from their wild type siblings in the amounts of time spent in the center of an open field or their latencies before emerging from a dark box (Table F in S2 Table; ANCOVA p = 0.216 and 0.263, respectively).

### Cocaine conditioned place preference

**Fig 2:**
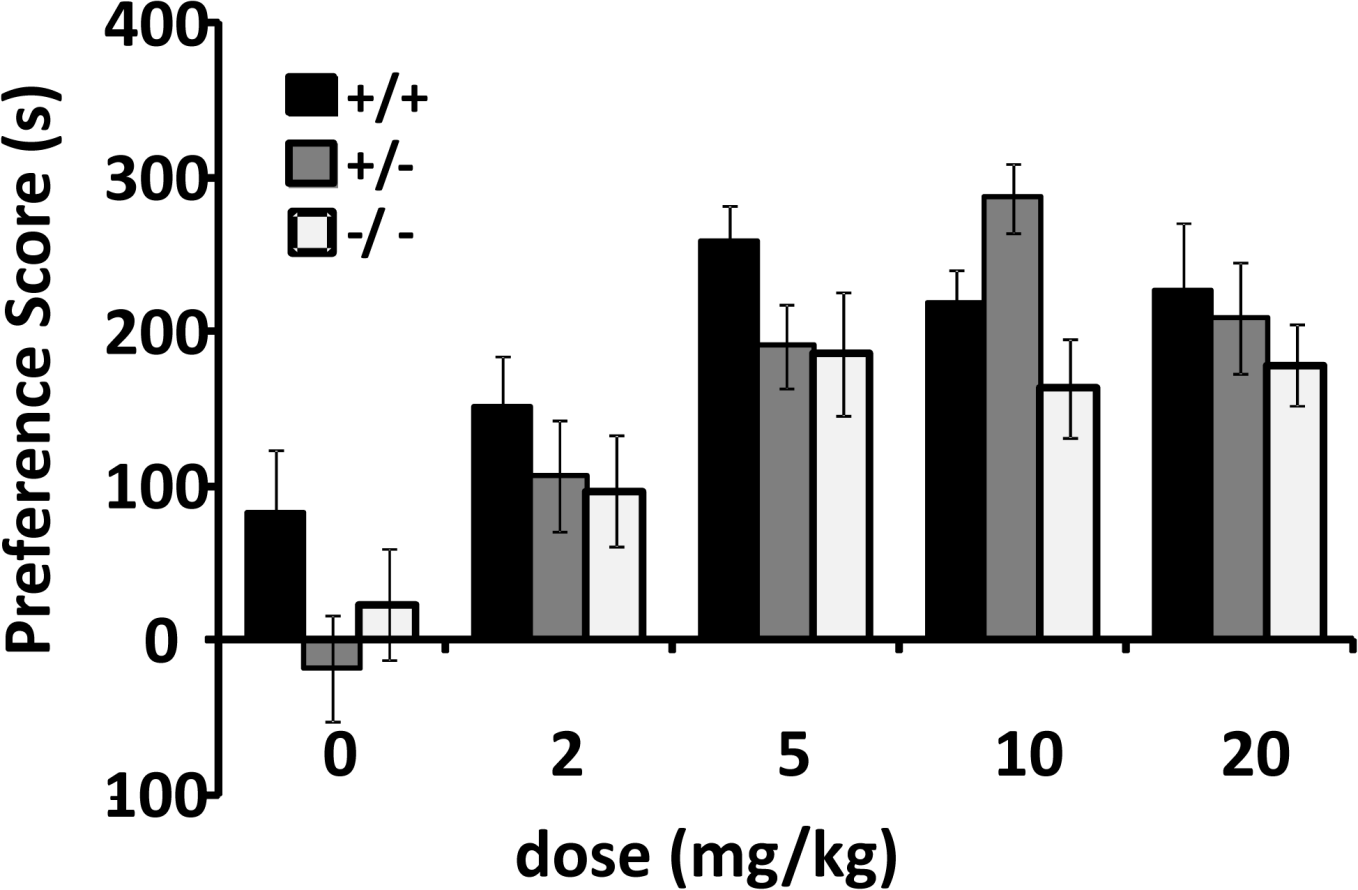
Cocaine conditioned place preference among mice with different CSMD1 genotypes. Mean difference ± SEM in time spent on the cocaine-paired side (Y axis) before and after conditioning with different doses of cocaine for wildtype heterozygous and homozygote knockout mice (ANCOVA effect of genotype p = 0.024; n = 13–26 mice of each genotype for each dose). Data from male and female mice are combined since gender displayed no significant interaction with genotype.

In wildtype mice, cocaine-conditioned place preference (CPP) was maximal at 5–10 mg/kg doses, as we and others have observed in studies of many other knockout mouse strains (Fig 2; Table G in S2 Table) [33]. There was a significant main effect of *csmd1* genotype (p = 0.024). At most doses, mice with reduced *csmd1* expression displayed numerically-reduced preferences for places where they had previously received cocaine. There was numerically-greater preference for places paired with 10 mg/kg cocaine in heterozygous knockouts. There was substantial variation among three subcohorts of mice (n = 7–11 each) that received this dose; there was p = 0.152 for genotype*dose interaction.

### Locomotion

**Fig 3:**
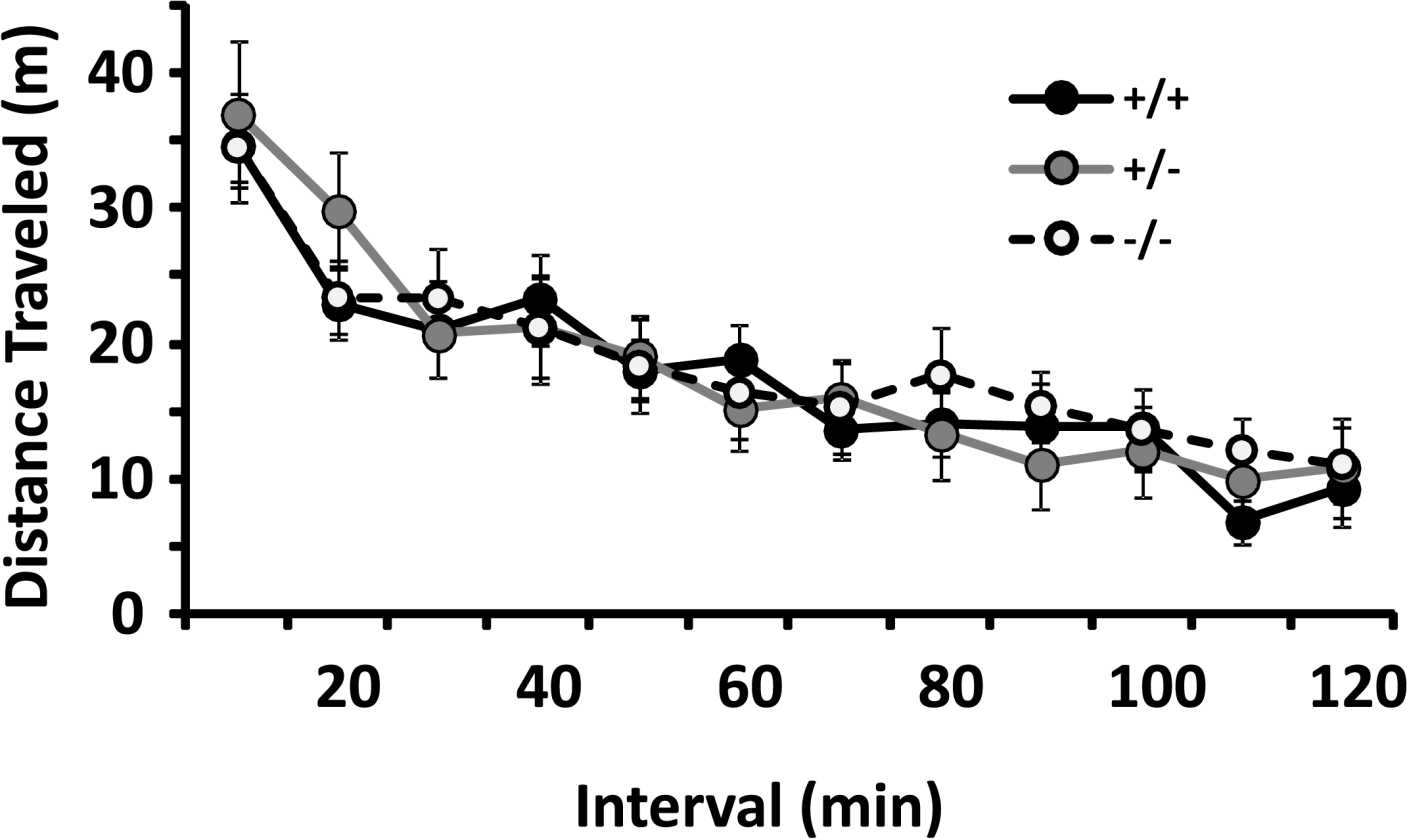
Locomotion and habituation in a novel 42 x 42 cm chamber: no significant differences between mice with different CSMD1 genotypes. Values are mean +/- SEM of the number of meters traveled when mice were exposed for the first time to the 42 x 42 cm apparatus (n = 10/genotype, ANOVA p = 0.967).

*Locomotion and locomotor habituation* measured in 42 x 42 cm dark and sound attenuated boxes to which the mice had not been previously exposed for 120 min trials did not identify significant influences of genotype (Fig 3; p for total distance traveled 0.967).

**Fig 4:**
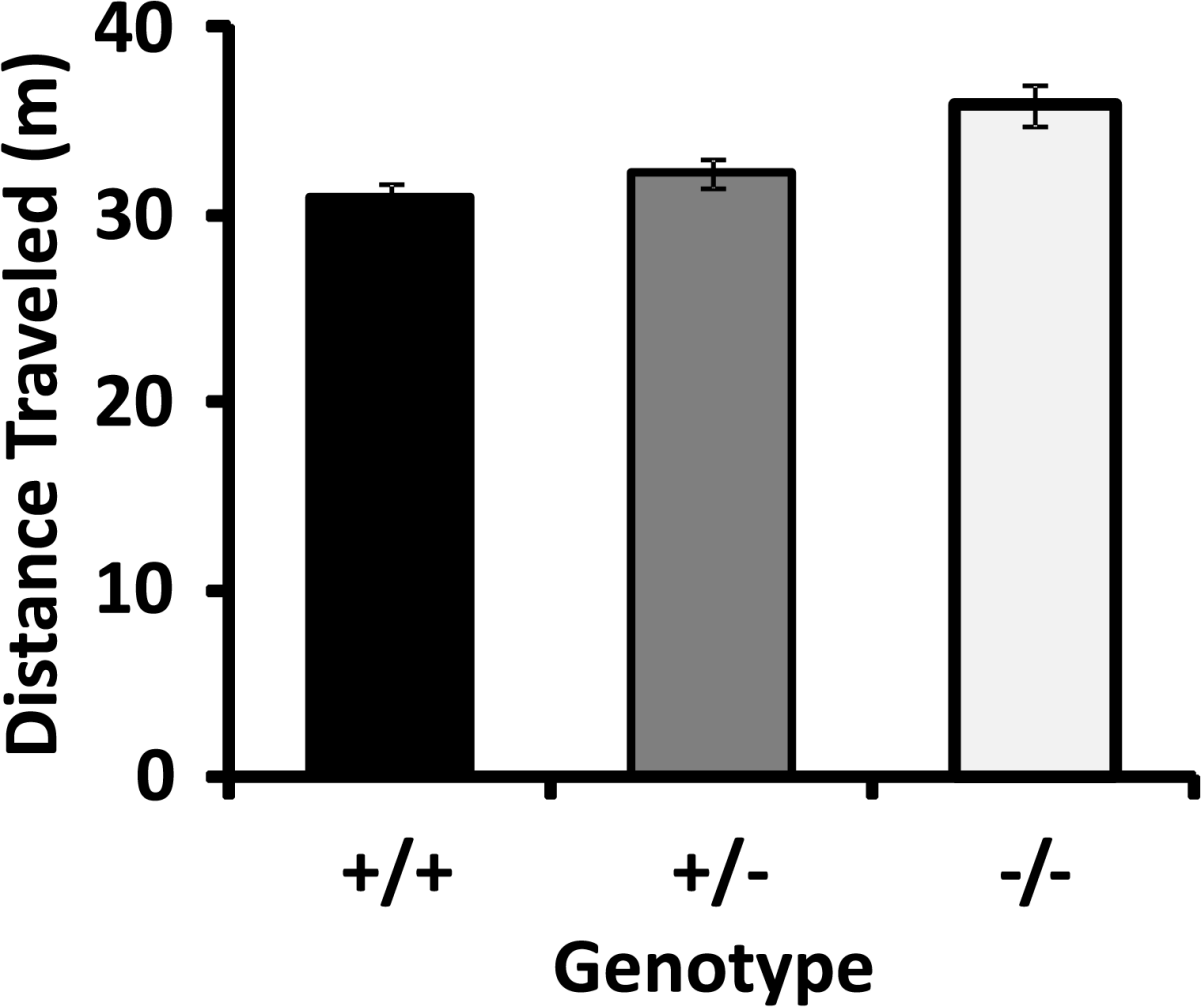
CSMD1 knockouts display modestly increased locomotion in a brightly lit environment. Values are mean +/- SEM of the number of cm traveled during the first pretest, in which mice were exposed for the first time to the CPP apparatus and allowed to explore both sides (n = 48/genotype, ANCOVA p = 3.7 x 10^−13^).

*Locomotion during baseline exposures to the CPP apparatus*. Mice were exposed to the CPP apparatus and allowed to explore both sides freely during the pretest that established baseline preference for each mouse. ANCOVA of data from all of the subjects revealed a highly-significant effect of genotype on locomotion during the first exposure to this smaller, more brightly lit novel environment (Fig 4; Table H in S2 Table; p = 3.7 x 10^−13^). Scheffe’s post-hoc testing showed striking significance for the differences between homozygotes and their wild-type siblings (p = 3.7 x 10^−12^) and a moderate difference between wild type and heterozygous mice (p = 0.045). There was a significant effect of gender (p = 1.0 x 10^−11^) but no significant genotype*sex interaction (p = 0.800).

**Fig 5:**
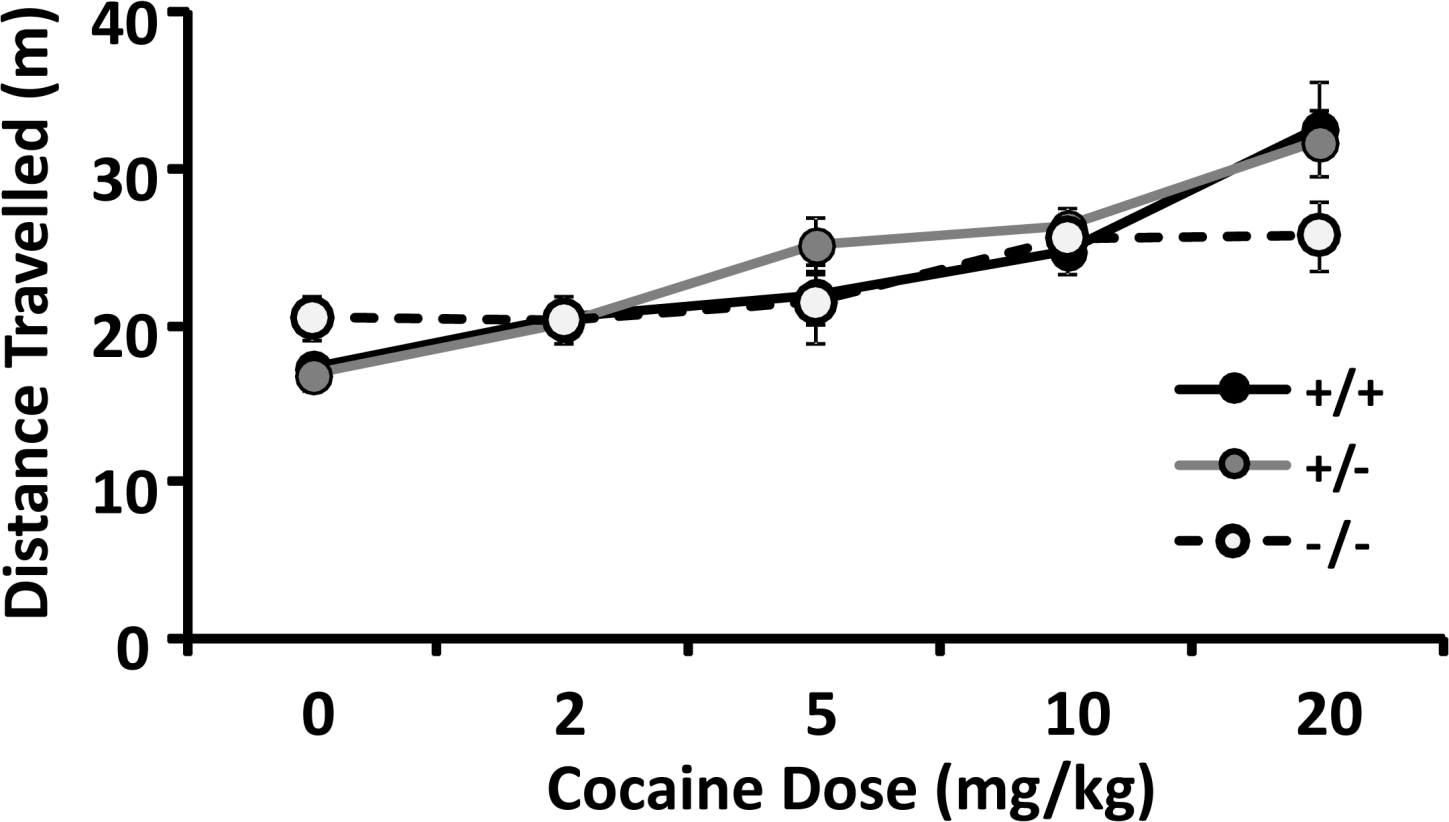
CSMD1 knockouts display differences in locomotion during the 20 min conditioning sessions when confined to 20 x 20 cm portions of the conditioned place preference boxes after receiving their first saline or cocaine injections. Saline-injected CSMD1 knockouts displayed significantly increased locomotion (p = 0.002) in comparison to mice of other genotypes. Significant effects of cocaine dose and gender were identified in ANCOVA of data from cocaine-injected mice of all genotypes (p = 5.5 x 10^−15^ and 0.009, respectively). There was a trend towards genotype * dose interaction (p = 0.054). Values are mean +/- SEM of the number of m traveled.

*Locomotion during the first 20 min conditioning session* was assessed when mice were confined to 20 x 20 cm portions of the conditioned place preference boxes after receiving their first saline or cocaine injections. Saline-injected *csmd1* knockouts displayed significantly increased locomotion (Fig 5; Table I in S2 Table; p = 0.002) in comparison to mice of other genotypes. Significant effects of cocaine dose and gender were identified in ANCOVA of data from cocaine-injected mice of all genotypes (p = 5.5 x 10^−15^ and 0.009, respectively). There was a trend towards genotype*dose interaction (p = 0.054). Cocaine injections increased locomotion in wild type and heterozygous mice (p = 4.7 x 10^−6^ and 4.9 x 10^−8^, respectively), but did not significantly increase locomotion in the homozygotes (p = 0.078).

*Locomotor sensitization* was sought by comparing locomotion during the second *vs* first cocaine conditioning sessions. Mice of different genotypes failed to display significant overall differences between the quantities of locomotion that they displayed during these two sessions (repeated measures ANCOVA effect of genotype (Table J in S2 Table; p = 0.432,). By contrast, there was a highly significant main effect of dose (p = 3.5 x 10^−26^), significant genotype*dose interactions (p = 0.007) and significant dose*session interactions (p = 2.0 x 10−^13^).

**Fig 6:**
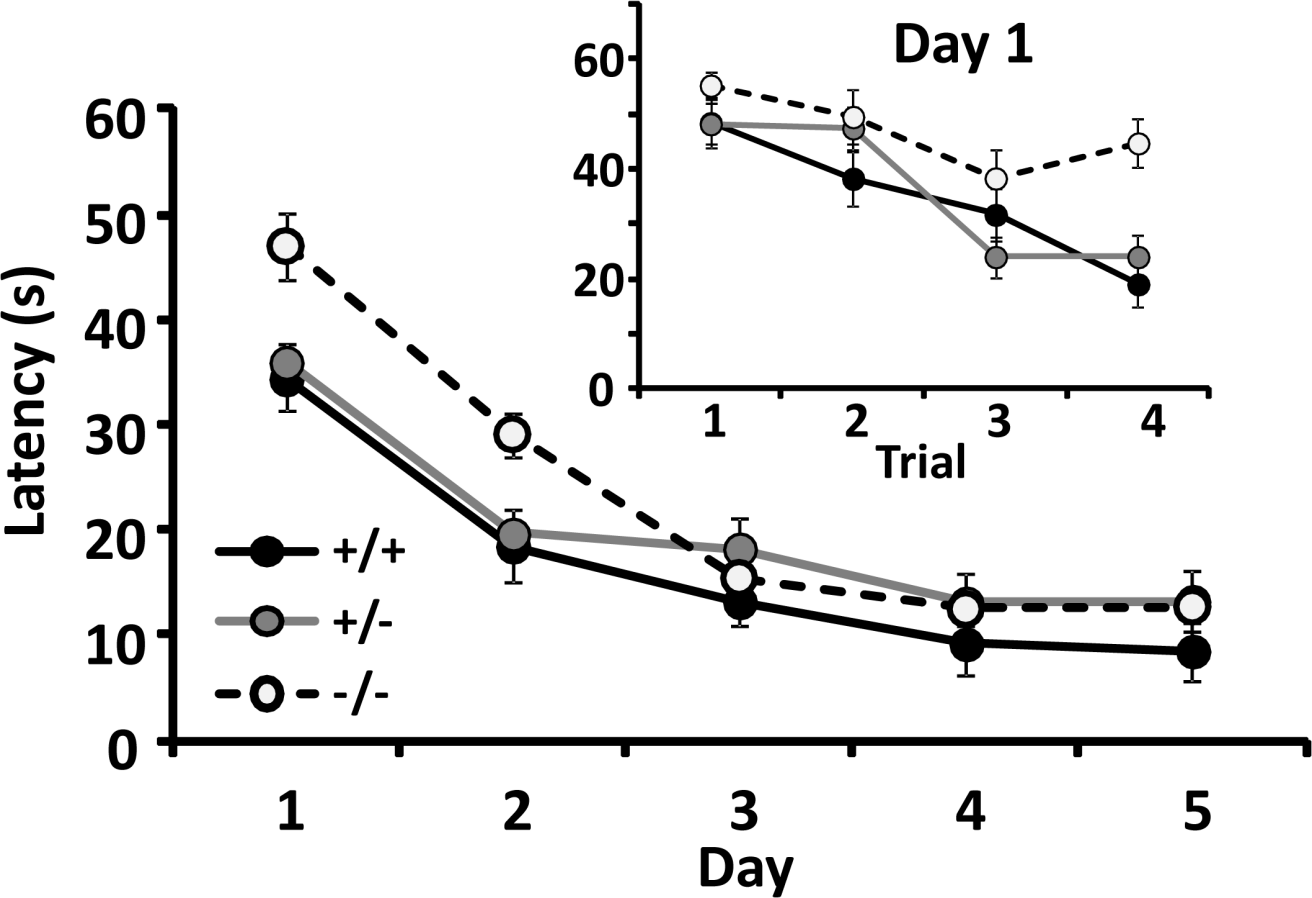
Morris water maze performance: time to reach platform in mice with different CSMD1 genotypes. *Main Fig*: Averaged daily latencies ± SEM to reach the Morris water maze platform for wildtype, heterozygous and homozygous CSMD1 knockout mice (repeated measures ANOVA genotype * day interaction p = 0.004 for learning. There were no significant differences in swimming speed (*see text*). *Inset*: More detailed data showing latencies to reach the (visible) platform during the first four trials (*day 1*). The trend for poorer performance in the homozygous knockouts reached the margin of statistical significance (p = 0.058).

*Memory*: *Morris water maze*: Mice of all *csmd1* genotypes swam through the Morris water maze apparatus with similar velocities (Table M in S2 Table; p = 0.106). Averaged daily latencies to reach the platform during the learning phase of the task were significantly longer in the homozygous *csmd1* knockouts (Fig 6; Table K in S2 Table; repeated measures ANOVA genotype*day interaction p = 0.004). Effects were especially notable during the first day of training (Fig 6 inset; Table L in S2 Table). Latencies during the first trial on this first day were similar across genotypes. Wild type and heterozygote knockouts improved performance during the day, but homozygote knockout mice failed to improve performance. These differences reached the margin of statistical significance (repeated measures ANOVA genotype * trial interaction p = 0.058). These results are consistent with at least some results from some of the studies of cognitive associations with human *CSMD1* variants [20,23,24] and cognitive tests of mixed-background *csmd1* knockouts reported from other laboratories [21,27]. Despite the longer latencies to find the hidden platform during the acquisition phase of testing, the homozygous *csmd1* knockouts did not display significantly worse performance during probe trials in which the platform was removed (Table M in S2 Table; distance from platform location p = 0.854, time in target quadrant p = 0.909).

## Discussion

Our current results document 15–25% differences in levels of expression of *CSMD1* in postmortem brains of individuals with common *CSMD1* genotype marker alleles that lie near *CSMD1* markers that have been associated with substance dependence, ability to quit smoking and vulnerability to developing schizophrenia. These nominally-significant associations do not reach Bonferroni-corrected statistical significance. In *csmd1* knockout mice with reduced CSMD1 mRNA expression, there is an overall alteration in preference for places associated with exposure to effects of cocaine. Taken together with data that describe association between human *CSMD1* variants and addiction-related phenotypes, these combined results support likely contributions of moderate individual differences in levels of CSMD1 expression to individual differences in levels of responses to rewarding and addictive drugs.

Conditioned place preference tests combinations of drug reward and the memory processes that are triggered by prior rewarding drug-associated experiences. It is thus conceivable that the reduced CPP in homozygous knockouts could receive contributions from the observed reductions in performance on mnemonic testing. Others’ results in which *csmd1* knockouts on mixed genetic backgrounds fail to display substantially altered memory in tests of novel object recognition or differences in sucrose preference fail to provide evidence for large confounding influences in these mice [21,27]. Alterations in cocaine-induced place preference in the heterozygous *csmd1* knockout mice that express little difference in mnemonic testing suggest that we cannot ascribe the CPP findings in these mice to mnemonic influences of more moderately-altered levels of *csmd1* expression, however.

The modest changes in Morris water maze performance in the *csmd1* homozygous knockout mice with virtual elimination of CSMD1 expression are consistent with other data from mice and humans. These results accord with the prominent CSMD1 expression in memory-associated brain regions, especially the hippocampus. They are consistent with a report of more frequent CSMD1 copy number variants in individuals with mild cognitive impairment or dementia, when compared to controls [25]. The overall data, and contrasting data for missense or other common variation, support the idea that variation in levels of CSMD1 expression attributable to 5’ haplotypes in this gene provides a significant molecular source of common functional human individual difference at the *CSMD1* locus. This positive set of translational data, some of which achieve only nominal statistical significance, contrasts with others’ inabilities to detect significant differences in prepulse inhibition in *csmd1* knockout animals with mixed genetic backgrounds [21,27]. Taken together, these data support the possibility that human CSMD1 variation might play disproportionate roles in schizophrenia vulnerabilities due to influences on the cognitive difficulties that are commonly experienced by individuals with this diagnosis [34], though recent analyses also point to roles for CSMD1 variation in core schizophrenic psychotic features and in disease severity, providing a caution to accepting this simple explanation [16].

The intron 5 SNP rs10503223 and the intron 3 trinucleotide repeat annotated as rs71534387 are each associated with modest-to-moderate individual differences in levels of expression of CSMD1 mRNA that provide results at the margins of Bonferroni-corrected statistical significance. Variants in 5’ aspects of many genes are associated with their levels of expression [35]. The relatively large set of brains available here for these “level of expression” association studies, the relatively 5’ location of the genomic markers that display nominally-significant association and the lack of common missense variants in the 5’ exons of this gene provide support for common level of expression variation in CSMD1. There are also features that suggest caution. As noted above, the observed SNP and SSLP associations with levels of expression fail to surpass Bonferroni corrections for the numbers of correlated markers tested. The SNP whose variants are most studied for association with schizophrenia and individual differences in cognitive function, rs10503253, fails to display significant association with levels of CSMD1 mRNA expression. Though rs10503253 lies only *ca* 35 kb from the rs71534387 SSLP marker that does display associations with levels of CSMD1 expression, there is only modest linkage disequilibrium (D’ = 0.1) between these markers in the individuals whose brains we sampled here. Indeed, there is substantial variation in the patterns and extents of linkage disequilibrium across the 5’ aspects of the *CSMD1* gene in samples from different populations (http://hapmap.ncbi.nlm.nih.gov/cgi-perl/gbrowse/hapmap3r2_B36/#search), supporting the idea that use of the markers described here will provide differing patterns of information about nearby variations in samples from distinct populations.

The differences in expression associated with these markers’ alleles suggest that heterozygous and/or homozygous knockout mice with reduced expression provide valid models for common variation in CSMD1 in ways that accord nicely with their manifestation of phenotypes relevant to addiction and cognitive abilities. Based on the human associations with addiction phenotypes, it is thus of interest both that there are significant differences in cocaine conditioned place preference in the heterozygous knockout mice and that these differences display some evidence for specificity. Heterozygous CSMD1 knockout mice fail to display reductions in tests of locomotion or swimming speed that would provide confounding explanations for altered performance in tests of preference for places paired with cocaine or the Morris water maze. There is no evidence that heterozygous knockout alters the conditioned locomotion that can be exerted by cocaine during conditioned place preference testing. There is no enhanced lethality or other profound physiological alteration noted with the lifelong reductions in CSMD1 expression found in heterozygous knockouts. Others have reported only modest changes in expression of genes other than CSMD1 in initial *csmd1* knockouts [21]. In *csmd1* mice, we have found only modest (25–30%) increases in cerebral cortical levels of expression of mRNAs for the two CSMD1 family members, CSMD2 and CSMD3 (ANOVA p = 0.015 and < 0.001, respectively), providing additional evidence for the modest magnitude of the adaptive brain changes caused by lifelong reductions in CSMD1 expression (*JD and GRU*, *unpublished observations*, *2013*).

The current results add to data from studies of mice with altered expression of other cell adhesion molecules (NrCAM [36], CDH13, PTPRD (JD, GRU *et al*, *submitted*) and receptors (nAChR5 [37]), providing increasingly-robust validation for conditioned place preference as a model for human allelic variation associated with addiction vulnerability phenotypes. The conditioned place preference results from studies of heterozygous *csmd1* knockouts add significant value in confirming and extending modest-magnitude human associations that might otherwise be difficult to confirm in any other manner. These data support the working hypothesis that differences in neuronal connections, including those mediated by differences in cell adhesion molecules, are likely to help underpin individual differences in addiction and cognitive phenotypes [38].

## Supporting Information

S1 Table Primer sequences used for SNP genotyping using Sequenom panels and for the simple sequence repeat annotated as rs71534387. (XLSX)

S2 Table Data and statistical analysis from mouse behavioral tests. Table A: Original spreadsheet and statistical analysis of the 60 min locomotion in the original CSMD1 line. Mice were injected with saline or 10 mg/kg s.c. cocaine, as indicated and immediately placed into dark, sound-attenuating locomotor boxes (42 x 42 cm). Total distance traveled was calculated from infrared beam breaks by an Optovarimax ATS System. Table B: Original spreadsheet and statistical analysis of the Rotarod test in the original CSMD1 line. Latencies (s) to fall off on each day are presented. Table C: Original spreadsheet and statistical analysis of the weights of the back-crossed CSMD1 mice. Table D: Original spreadsheet and statistical analysis of the hanging wire test of the back-crossed CSMD1 mice. Table E: Original spreadsheet and statistical analysis of the Rotarod test of the back-crossed CSMD1 mice. Latencies (s) to fall off on each day are presented. Table F: Original spreadsheet and statistical analysis of the Dark box emergence and the open field tests of the back-crossed CSMD1 mice. For the Dark box emergence test, the testing cage (18 x 36 cm) consisted of two compartments: a dark chamber (18 x 18 cm) with black walls and a small opening (5 cm) leading to a Plexiglas compartment. An animal was placed into the dark chamber. The latency to emerge and the time spent outside the dark chamber during the 10 min trial were measured using the Optovarimax system (Columbus Instruments, OH). For the Open Field test, mice were placed singly in an open field (42 x 42 cm) for ten minutes. The time each animal spent in the central quadrant was recorded. Table G: Original spreadsheet and statistical analysis of the Conditioned Place preference (CPP) test of the back-crossed CSMD1 mice. Times spent on the cocaine-paired side during two pre-tests (Wire-T1, Wire-T2) and post-test (Wire-T3) and the difference (Ave ∆T) between the post-test and average of the two pretests (Ave T 1–2) are presented. Table H: Original spreadsheet and statistical analysis of the locomotion recorded during the CPP pre-test (T1- Loco) of the back-crossed CSMD1 mice. Table I: Original spreadsheet and statistical analysis of the locomotion recorded during the first CPP conditioning session (1st COC Loco) with the back-crossed CSMD1 mice. Table J: Original spreadsheet and statistical analysis of the locomotor sensitiziation of the back-crossed CSMD1 mice that occurred during the two CPP conditioning sessions (1st COC, 2nd COC). Table K: Original spreadsheet and statistical analysis of the Morris water maze (MWM) learning curves of the back-crossed CSMD1 mice. Values for each day are averages of four trials performed on that day. Table L: Original spreadsheet and statistical analysis of the Morris water maze (MWM) Latencies to reach the platform during each trial on day 1 are presented. Table M: Original spreadsheet and statistical analysis of the results from the (MWM) probe trial.
(XLSX)
